# Association of Complement C5 Gene Polymorphisms with Proliferative Diabetic Retinopathy of Type 2 Diabetes in a Chinese Han Population

**DOI:** 10.1371/journal.pone.0149704

**Published:** 2016-03-02

**Authors:** Dengfeng Xu, Hong Yi, Shizhi Yu, Xiaosong Li, Yanbin Qiao, Weiwei Deng

**Affiliations:** 1 Chongqing General Hospital, Chongqing EYE and ENT Hospital, Chongqing, P R China; 2 Chongqing Center for Clinical Laboratory, Chongqing Municipal People's Hospital, Chongqing, P R China; 3 Chongqing Three Gorges Central Hospital, Chongqing, P R China; Van Andel Research Institute, UNITED STATES

## Abstract

**Purpose:**

To investigate the association of C5 SNPs with proliferative diabetic retinopathy (PDR) of type 2 diabetes (T2D).

**Methods:**

A total of four C5 SNPs including rs2269067, rs7040033, rs1017119 and rs7027797 were genotyped in 400 PDR patients with T2D (cases) and 600 non- proliferative diabetic retinopathy PDR (NPDR) with T2D patients (controls) by using PCR-RFLP method. mRNA expression was examined by real-time PCR. Cytokine production was detected by ELISA.

**Results:**

The frequency of GG genotype of C5 rs2269067 was significantly increased in cases compared with controls (Pc = 3.4×10^−5^, OR = 1.87). And C5 mRNA expression was significantly increased in rs2269067 GG cases as compared with CG or CC cases (P = 0.003, P = 0.001, respectively). Moreover, the production of IL-6 was significantly increased in rs2269067 GG cases compared to CG cases or CC cases (P = 0.002, P = 0.001, respectively).

**Conclusions:**

C5 rs2269067 GG genotype confers risk for PDR of T2D in Chinese han population and is associated with an elevated C5 mRNA expression and an increased IL-6 production.

## Introduction

Diabetic retinopathy (DR) is a frequent complication of type 2 diabetes. Proliferative diabetic retinopathy (PDR), characterized as the advanced complication of T2D, is one of the principal eye diseases leading to blindness in working-age adults all over the world[1]. Previous studies show that neovascularization induced by ischemia is a pathological marker of PDR. Moreover, vitreous hemorrhage and tractional retinal detachment caused by neovascularization often lead to irreversible vision loss[2, 3]. Moreover, many studies have comfirmed the association of PDR with immune-related factors such as IL-17, IL-6, MCP-1, etc[4, 5].

The complement system is considered to play a critical role in the innate immune system[6]. Previous studies demonstrate that certain genetic variants of C5 are a risk factor for several immune related disorders, such as rheumatoid arthritis (RA) and systemic lupus erythematosus (SLE)[7–12]. Many studies have shown an association of C5 polymorphisms with age related macular degeneration (AMD), characterized as one kind of immune-mediated diseases[13–15].

Furthermore, many studies show that the complement system plays an important role in the initiation and development of PDR[16, 17]. Muramatsu et al[16] found that C5a concentration is significantly increased in vitreous from patients with PDR and is associated with an upregulated expression of VEGF and MCP-1. They suggest that C5a may play a key role in the pathogenesis of PDR in combination with inflammatory cytokines such as VEGF and MCP-1. Gao et al[18] observed a higher level of C3 protein in the vitreous of PDR patients as compared to the virtreous of nondiabetic individuals.

Although the exact etiology and mechanism of PDR is unclear, a large number of studies have demonstrated that genetic variants are critical to the pathogenesis of PDR[19–21]. To the best of our knowledge, the association of C5 genetic variants with PDR has not yet been addressed and was therefore the subject of this study.

## Methods

### Study design

A total of 400 PDR patients with T2D and 600 NPDR patients with T2D were enrolled in this study. All the individuals were Chinese Han. Blood samples were taken from patients visiting Chongqing Municipal People's Hospital and Chongqing three gorges central hospital (Chongqing, China). The diagnosis of diabetes was based on standard criteria defined by the American Diabetes Association[22]. The detailed eye examinations were performed by using the Early Treatment of Diabetic Retinopathy Study (ETDRS) protocol with seven-standard-field stereoscopic fundus photography. Retinopathy status was evaluated by fundus photographs and graded based on clinical ETDRS criteria. Patients with any disk neovascularization, neovascularization elsewhere, fibrovascular proliferation, vitreous hemorrhage, or tractional retinal detachment were defined as having PDR. Retinopathy grading was carried out without prior knowledge of genotypes. The individuals have similar duration of T2D and level of glycated hemoglobin. Duration of T2D and level of glycated hemoglobin are similar across all individuals included in the study.

### Ethics statement

The study was approved by the Ethics Research Committee of Chongqing Municipal People's Hospital. All procedures in this study were based on the principles of the Declaration of Helsinki. A written informed consent was obtained from every participant before collecting samples of peripheral venous blood. All methods were performed in accordance with the approved guidelines.

### SNP selection

Based on previous association studies of certain immune-related diseases, ten C5 SNPs (rs2269067, rs17611, rs7026551, rs10985126, rs7037673, rs1468673, rs7040033, rs2269066, rs25681, rs7027797) were initially selected as candidates for this study[12, 14, 15, 23–25]. Two groups of SNPs were found to be in linkage disequilibrium, SNPs rs7026551, rs2269066, rs10985126, rs1468673 and rs2269067 (r^2^≥0.86) (S1 Fig) and SNPs rs7037673, rs17611, rs25681, rs7040033 (r^2^ = 1) (S2 Fig). Thus, three C5 SNPs (rs2269067, rs7040033, and rs7027797) plus a TagSNP (rs1017119) were examined (S1 Table). Details of C5 SNPs characteristics are shown in S1 Table.

### DNA extraction and SNP genotyping

Genomic DNA was extracted from peripheral blood using the Qiagen QIAamp DNA Mini blood kit (Qiagen, California, USA). Genotyping was examined by PCR-RFLP method. The primer sequences and restriction enzymes are shown in S2 Table. Four percent agarose gels were used to separate the digestion products and colored with GoldView (SBS Genetech, Beijing, China). Data was analyzed to make sure that the study population was in Hardy-Weinberg equilibrium (HWE). Ten percent of study samples were randomly selected for direct sequencing to assure the validity and 100% concordance of the results of the PCR-RFLP with direct sequencing of the SNP genotyping.

### Cell isolation and culture

Peripheral blood mononuclear cells (PBMCs) from PDR patients with T2D were isolated using Ficoll-Hypaque density-gradient centrifugation. The isolated PBMCs were then seeded in 24-well plates (2×10^6^ cells per well) and cultured for 24 hours or 72 hours in 100μg/ml streptomycin, RPMI medium 1640 supplemented with 10% fetal calf serum and 100U/ml penicillin. PBMCs were stimulated with a mixture of anti-CD3 (5μg/ml, eBioscience, San Diego, CA, USA) and anti-CD28 antibodies (1μg/ml, eBioscience, USA) for 72 hours to test the production of IL-10 in the culture supernatants. To detect the level of IL-1β, IL-8, TNF-α, IL-6 and MCP-1, PBMCs were stimulated with 100ng/ml lipopolysaccharide (100ng/ml, Sigma-Aldrich, St. Louis, MO, USA) for 24 hours and then the supernatants were collected for cytokine measurements.

### Real-time PCR

TRIzol (Invitrogen, Carlsbad, CA, USA) and transcriptase kit (Applied Biosystems, Foster City, California, USA) were used for total RNA extraction and cDNA reverse transcription, respectively. The primer sequences for C5 were as follows: forward: 5’-GGCACAAAGTCCTCCAAATG-3’, reverse: 5’-CCAAACCAAGTCTCCAGTG A-3’. GAPDH was chosed as the internal reference gene and the 2^−ΔΔCt^ method was used to calculate the relative C5 mRNA expression.

### ELISA

The human Duoset ELISA development kit (R&D Systems, Minneapolis, MN, USA) was used to measure the concentration of IL-6, MCP-1, IL-8, IL-10, TNF-α and IL-1β in PBMC culture supernatants based on the manufacturer’s protocols.

### Statistical analysis

Chi-square test (SPSS version 17.0, Inc., Chicago, Illinois, USA) was used to compare the frequencies of genotypes and alleles between cases and controls. The levels of mRNA and various cytokines were analyzed by one-way ANOVA among three different genotypes. Bonferroni correction was used for multiple comparisons.

## Results

### Clinical characteristics of T2D patients with or without PDR

Clinical details, including age and gender, are shown in S3 Table.

### Increased frequency of C5 GG genotypes in patients with PDR

The four SNPs were genotyped in 400 PDR patients and 600 NPDR patients. The frequencies of all the genotypes showed no deviation from HWE. Our results showed that the GG genotype and the G allele frequency of rs2269067 were significantly increased in cases compared with controls (Pc = 3.4×10^−5^, OR = 1.87 and Pc = 5.6×10^−4^, OR = 1.52, respectively) (Table 1). The genotype and allele frequencies of the other three SNPs showed no statistical difference between cases and controls (Table 1).

**Table 1:** Frequencies of genotypes and alleles of C5 polymorphisms in PDR patients with T2D and NPDR patients with T2D.

SNPs	Allele	PDR (%)	NPDR (%)	P value Pc OR(95%CI)		
rs2269067	G	645 (80.6)	879 (73.2)	1.4×10^−4^	5.6×10^−4^	1.52 (1.22–1.88)
	CC	30 (7.5)	45 (7.5)	1.000	NS	1.00 (0.62–1.61)
	CG	95 (23.7)	231 (38.5)	1.0×10^−6^	1.1×10^−5^	0.49 (0.37–0.66)
	GG	275 (68.8)	324 (37.0)	3.1×10^−6^	3.4×10^−5^	1.87 (1.43–2.44)
rs7040033	A	475 (59.3)	708 (59.0)	0.867	NS	1.01 (0.84–1.21)
	AA	135 (32.4)	204 (32.2)	0.935	NS	0.98 (0.75–1.29)
	AG	205 (53.5)	300 (52.2)	0.699	NS	1.05 (0.81–1.35)
	GG	60 (14.1)	96 (15.6)	0.669	NS	0.92 (0.65–1.31)
rs1017119	C	124 (15.5)	215 (17.9)	0.158	NS	0.84 (0.66–1.07)
	CC	15 (3.7)	30 (5.0)	0.350	NS	0.74 (0.39–1.39)
	CT	94 (23.5)	155 (25.8)	0.403	NS	0.88 (0.65–1.18)
	TT	291 (72.8)	415 (69.2)	0.223	NS	1.19 (0.89–1.57)
rs7027797	C	75 (9.3)	90 (7.5)	0.135	NS	1.27 (0.92–1.75)
	CT	75 (18.7)	90 (15.0)	0.118	NS	1.31 (0.93–1.83)
	TT	325 (81.3)	510 (85.0)	0.118	NS	0.76 (0.54–1.07)

T2D: Type 2 diabetes; PDR: Proliferative diabetic retinopathy; NPDR, non-proliferative diabetic retinopathy; SNP, single-nucleotide polymorphism; OR, odds ratio; CI, confidence interval; NS, non-significant; Pc, P Bonferroni correction.

### Increased C5 mRNA expression in PDR patients with rs2269067 GG genotype

**Fig 1:**
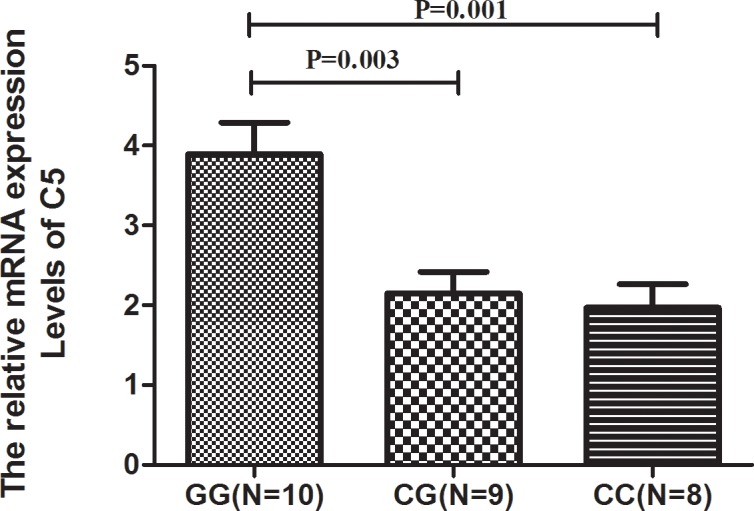
The mRNA expression of C5 in different genotype cases. mRNA expression of C5 in PBMCs from PDR patients with T2D carrying different genotypes of rs2269067 (GG: n = 10, CG: n = 9, CC: n = 8). Significance was analyzed by SPSS one-way ANOVA. Data are shown as mean±SD (Standard deviation).

The aforementioned results demonstrated a significant association of rs2269067 with PDR. We then investigated the C5 mRNA expression in PBMCs from PDR patients with T2D carrying a known SNP genotype. The C5 mRNA expression of rs2269067 GG genotype cases was significantly increased as compared to CG and CC cases (P Bonferroni = 0.003, P = 0.001, respectively) (F_sig_ = 0.001) (Fig 1).

### The influence of C5 rs2269067 on cytokine production

**Fig 2:**
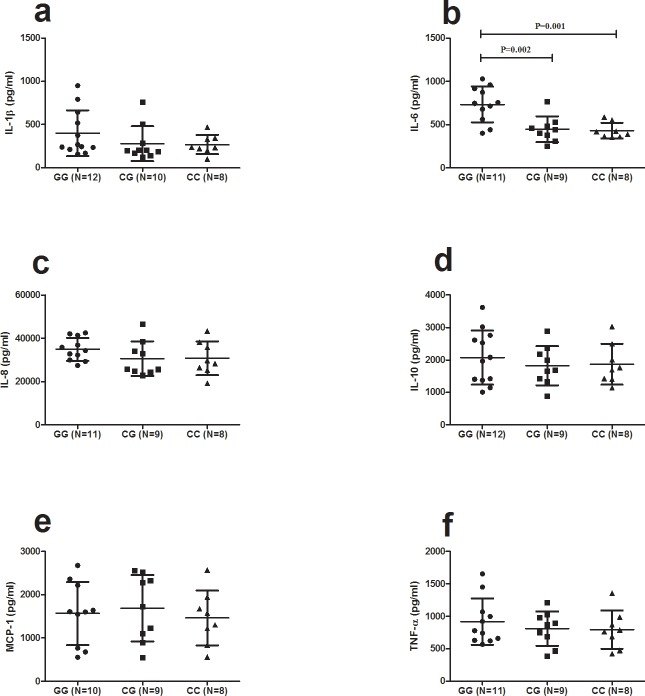
The influence of C5 rs2269067 on cytokine production. Cytokine production by anti CD3/CD28 or LPS stimulated PBMCs from PDR patients with T2D carrying different genotypes of rs2269067 (GG: n = 10–12, CG: n = 9–10, CC: n = 8). Significance was was analyzed by SPSS one-way ANOVA. Data are shown as mean±SD (Standard deviation).

A further experiment was performed to test whether the three different genotypes of rs2269067 affected cytokine production. The level of IL-6, IL-10, IL-8, IL-1β, TNF-α and MCP-1 was examined in the culture supernatants of stimulated PBMCs from genotyped PDR patients. The results showed that the level of IL-6 in rs2269067 GG cases was significantly higher than that in CG cases and CC cases (P Bonferroni = 0.002, P Bonferroni = 0.001, respectively) (F_sig_ = 3.6×10^−4^) (Fig 2). There was no association between the other five cytokines and rs2269067.

## Discussion

In the present study, we investigated the association of four C5 SNPs with PDR of T2D in a Chinese Han population. The results showed that C5 rs2269067 was a risk factor for PDR with T2D. Functional studies showed that C5 mRNA expression in the rs2269067 GG genotype cases was increased as compared to the other two genotype cases. Additionally, a significantly increased production of IL-6 was observed in PBMCs stimulated by LPS from rs2269067 GG genotype cases.

A large number of studies have shown the association of PDR with immune response[26, 27]. In one study, high concentrations of inflammatory cytokines such as IL-6 and TNF-a were observed in vitreous of T2D patients with PDR[27]. This observation suggests that the inflammatory-immune process is critical in the pathogenesis of PDR. Consistent with the aforementioned study, Canataroglu et al[26] found that the levels of IL-6 and IL-8 were elevated in vitreous of patients with PDR or with proliferative vitreoretinopathy (PVR) and suggested that IL-6 and IL-8 may play an important role in the pathogenesis of PDR and PVR.

Complement C5 has been regarded as a critical element in immune related disorders[28, 29]. Kessel et al[28] found that anti-C5a treatment could significantly alleviate the severity of arthritis by inbibiting the production of inflammatory cytokines including IFN-γ in mouse models of experimental arthritis. Nozaki et al[30] demonstrated that knockout of the receptors of C3a or C5a could both significantly reduce the expression of VEGF and formation of choroidal neovascularization after laser injury in mouse models of age-related macular degeneration (AMD). Moreover, a significantly decreased choroidal neovascularization was observed in this model by blocking of C3a or C5a receptor.

In our study we found an association of C5 SNPs with IL-6, but C5 SNPs were not associated with other cytokines including IL-1β, IL-8, IL-10, MCP-1 or TNF-α. Although the approach we chose (LPS stimulation of genotype PBMCs) did not find an association of C5 SNPs with proinflammatory cytokines we cannot exclude that the various C5 genetic variants may affect proinflammatory cytokine production via other pathways. Moreover, further studies are needed to confirm the exact mechanism of these genotypes on the inflammatory response. Notably, examination of direct cytokine assays is also important and needs to be further studied.

Few studies have shown an association of C5 polymorphisms with diabetic disorders. To the best of our knowledge, the association between genetic variants of C5 and PDR has not yet been reported.

How C5 SNP rs2269067 affects the risk for PDR is not yet clear. Preliminary data showed that C5 mRNA expression was enhanced in the rs2269067 GG genotype cases. We further showed that IL-6 was elevated in rs2269067 GG genotype cases. It suggests that the C5 GG rs2269067 genotype may increase the production of IL-6 through C5 mRNA expression, thereby influencing the development of PDR.

Of note, the lower mRNA expression and IL-6 response in the CG genotype would fit in with the protection against disease in the patients of PDR with this genotype. Contrary to the result of our study, a recent study showed that C5 blockade or C5aR deficiency could enhance pathological retina angiogenesis in mouse models[31]. Although the reasons for this discrepancy are unclear, differences between humans and mice might be one of the explanations. Noticeably, our results are, by and large, consistent with those reported in AMD patients and AMD mouse models, in which activation of C5a could induce retina angiogenesis or ablation of C5aR could reduce retina angiogenesis, respectively[30]. Moreover, consistent with previous studies, our results showed that upregulated C5 expression was associated with a higher level of IL-6 in PBMCs[5, 32, 33]. Whether modulation of C5 could have therapeutic implications in our PDR patients therefore also deserves further basic investigation. Of interest is that anti-C5 therapy has been shown to alleviate the severity of disease in mouse models of experimental induced uveitis[34].

There are some limitations that should be taken into account in this study. Although our results showed that C5 mRNA was significantly different in PBMCs from PDR patients with different genotypes of rs2269067, whether the level of C5 protein was consistent with the result of C5 mRNA is unclear. Moreover, how the rs2269067 would affect C5 activity remains unknown and should be studied in the future. This study was performed only in Chinese Han population, and replication in an independent sample would strengthen our findings. Our findings also must be confirmed in other ethnic populations. The PDR patients were all recruited from T2D in this study. Whether the association of C5 polymorphisms with PDR of type 1 diabetes remains unclear and needs to be further studied. In addition, whether other non-diabetic proliferative retinopathy including central retinal vein occlusion (CRVO), rhegmatogenous retinal detachment, ocular penetrating injury showed an association with C5 polymorphisms remains unclear and future study needs to be investigated.

In conclusion, our results revealed that the C5 rs2269067 confers a risk for PDR with T2D in a Chinese Han population. The risk genotype affects the mRNA expression of C5 and the production of proinflammatory cytokines such as IL-6.

## Supporting Information

S1 Fig (DOC)

S2 Fig (DOC)

S1 Table (DOC)

S2 Table (DOC)

S3 Table (DOC)
