# Supplementary figures and images for: Association of Complement C5 Gene Polymorphisms with Proliferative Diabetic Retinopathy of Type 2 Diabetes in a Chinese Han Population
Source: PLoS One. 2016 Mar 2;11(3):e0149704. doi: 10.1371/journal.pone.0149704 (PMC4775016; doi:10.1371/journal.pone.0149704)

Supplementary Figure 1 The C5 linkage disequiliburium plot


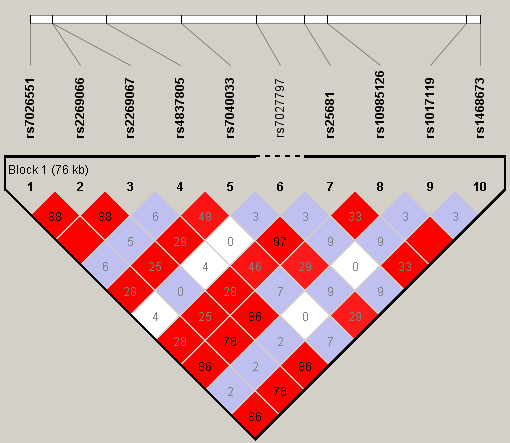

Supplement: S1 Fig — (DOC) [file pone.0149704.s001.doc]
