# Supplementary material for: Association of Complement C5 Gene Polymorphisms with Proliferative Diabetic Retinopathy of Type 2 Diabetes in a Chinese Han Population
Source: PLoS One. 2016 Mar 2;11(3):e0149704. doi: 10.1371/journal.pone.0149704 (PMC4775016; doi:10.1371/journal.pone.0149704)
Supplement: S2 Fig — (DOC) [file pone.0149704.s002.doc]

Supplementary Figure 2


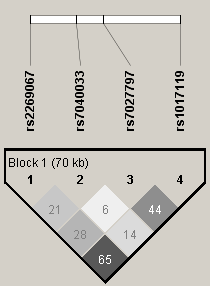


Pair-wise linkage disequilibrium values of C5 SNPs in a Chinese Han population.

Linkage Disequilibrium (LD) block was estimated for C5 gene locus using our data.

The pair-wise r^2^ Values are shown in blocks.
