# Supplementary material for: Association of Complement C5 Gene Polymorphisms with Proliferative Diabetic Retinopathy of Type 2 Diabetes in a Chinese Han Population
Source: PLoS One. 2016 Mar 2;11(3):e0149704. doi: 10.1371/journal.pone.0149704 (PMC4775016; doi:10.1371/journal.pone.0149704)
Supplement: S1 Table — (DOC) [file pone.0149704.s003.doc]

Supplementary Table 1 Details of C5 SNPs characteristics

| SNP | Location | MAF | Disease reported |
| --- | --- | --- | --- |
| rs2269067 | 120974762 | C=0.3057 | Rheumatoid Arthritis, Behcet's disease, |
| rs7040033 | 120996766 | A=0.4519 | Rheumatoid Arthritis |
| rs1017119 | 121045260 | C=0.0509 | * |
| rs7027797 | 121009521 | C=0.0885 | Rheumatoid Arthritis |

MAF, minor allele frequency. *, SNP rs1017119 was selected from a TagSNP. No association of rs1017119 with immune related disease in previous studies.
