# Supplementary material for: Association of Complement C5 Gene Polymorphisms with Proliferative Diabetic Retinopathy of Type 2 Diabetes in a Chinese Han Population
Source: PLoS One. 2016 Mar 2;11(3):e0149704. doi: 10.1371/journal.pone.0149704 (PMC4775016; doi:10.1371/journal.pone.0149704)
Supplement: S2 Table — (DOC) [file pone.0149704.s004.doc]

Supplementary Table 2 Primers and restriction enzymes used for restricted fragment length polymorphisms analysis of C5 gene.

| **Gene** | **SNPs** | **Primer** | **Restriction enzyme** |
| --- | --- | --- | --- |
| **C5** | rs2269067 | 5’ ggcccctctgtacttccatgt 3’  5’ gccagtagaggtaaatgaagcac 3’ | DraIII-HF |
|  | rs7040033 | 5’ cacacatggaaatcaagtaac 3’  5’ aaattcacttcagtaaacaggta 3’ | Acc65I |
|  | rs1017119 | 5’ ccgccttctgggttcaa 3’  5’ aagcataagattgtaccgtttt 3’ | NspI |
|  | rs7027797 | 5’ AGGGCAGGGAGAGGAATACAA 3’  5’ TGCCCTCTTCAATGTCTCTTTCA 3’ | FokI |
