# Supplementary material for: Association of Complement C5 Gene Polymorphisms with Proliferative Diabetic Retinopathy of Type 2 Diabetes in a Chinese Han Population
Source: PLoS One. 2016 Mar 2;11(3):e0149704. doi: 10.1371/journal.pone.0149704 (PMC4775016; doi:10.1371/journal.pone.0149704)
Supplement: S3 Table — (DOC) [file pone.0149704.s005.doc]

Supplementary Table 3 Clinical details in T2D patients with PDR and T2D patients without PDR

| **Characteristic** | **PDR** | **NPDR** | ***P* value** |
| --- | --- | --- | --- |
| Age （mean±SD） | 59.5±0.5 | 60.2±0.4 | NS |
| Gender (male/female) | 174/226 | 253/347 | NS |
| Duration (years) | 13±0.3 | 14±0.3 | NS |
| HbA1c (mg/dl) | 9.0±0.1 | 8.9±0.2 | NS |
| BMI (kg/m^2^) | 25.1±0.2 | 24.8±0.2 | NS |

T2D : Type 2 diabetics; PDR: Proliferative diabetic retinopathy; HbA1c: Glycosylated hemoglobin; BMI: Body mass index.
